# Notification that new names of prokaryotes, new combinations and new taxonomic opinions have appeared in volume 75, part 11 of the IJSEM

**DOI:** 10.1099/ijsem.0.007010

**Published:** 2026-02-27

**Authors:** Aharon Oren, Markus Göker

**Affiliations:** 1 The Institute of Life Sciences, The Hebrew University of Jerusalem, The Edmond J. Safra Campus, 9190401 Jerusalem, Israel; 2 Leibniz Institute DSMZ – German Collection of Microorganisms and Cell Cultures, Inhoffenstrasse 7B, 38124 Braunschweig, Germany

This listing of names of prokaryotes published in a previous issue of the *IJSEM* is provided as a service to microbiology to assist in the recognition of new names and new combinations. This procedure was proposed by the Judicial Commission [1]. The names given herein are listed according to the Rules of priority (i.e. time and date of publication of the articles on the journal’s platform and the order of valid publication of names in the original articles) [2].

**Table:** 

Order of article publication	Name/authors	Proposed as	Article no.	Page no.
1	*Endobacterium panacihumi* (Kang *et al.* 2021) Menéndez *et al.* 2025	comb. nov. [basonym: *Rhizobium panacihumi* Kang *et al.* 2021]	006945	4
2	*Foliimonas* Banerjee *et al.* 2025	gen. nov.	006953	10
2	*Foliimonas ilicis* Banerjee *et al.* 2025	sp. nov.	006953	11
3	*Pantoea graminicola* Ferreira *et al.* 2025	sp. nov.	006952	9
4	*Hymenobacter weizhouensis* Luo *et al.* 2025	sp. nov.	006940	6
4	*Hymenobacter latericius* corrig. Luo *et al.* 2025*	sp. nov.	006940	6
5	*Chryseobacterium chendengshani* Chen *et al.* 2025	sp. nov.	006941	10
5	*Chryseobacterium wangxinyae* Chen *et al.* 2025	sp. nov.	006941	11
6	*Mumia spirodelae* Vitoonpanyakit *et al.* 2025	sp. nov.	006961	7
7	*Caballeronia eucalypticola* Xu *et al.* 2025	sp. nov.	006955	6
8	*Pseudomonas tussilaginis* Juszczyk *et al.* 2025	sp. nov.	006968	11
9	*Mycobacterium camsae* Mei *et al.* 2025	sp. nov.	006960	4
9	*Mycobacterium pumcae* Mei *et al.* 2025	sp. nov.	006960	6
10	*Caloranaerobacter longqiensis* Li *et al.* 2025	sp. nov.	006962	8
11	*Tenacibaculum salmonis* Avendaño-Herrera *et al.* 2025	sp. nov.	006963	9
12	*Apilactobacillus intestinapis* Tsai *et al.* 2025	sp. nov.	006971	4
13	*Ferrigenium straubiae* Becker and Kappler 2025	sp. nov.	006949	20
14	*Amycolatopsis rhizosphaerihabitans* Chen *et al.* 2025	sp. nov.	006974	8
15	*Bradyrhizobium monzae* Morel Revetria *et al.* 2025	sp. nov.	006973	5
16	*Microbaculum mangrovi* Huang *et al.* 2025	sp. nov.	006972	8
17	*Puribacter* Hatamoto 2025	gen. nov.	006978	6
17	*Puribacter membranae* Hatamoto 2025	sp. nov.	006978	7
18	*Dryocola clanedunensis* subsp. *clanedunensis* (Maddock *et al.* 2023) Menasria *et al.* 2025	comb. nov. [basonym: *Dryocola clanedunensis* Maddock *et al.* 2023]	006977	8
18	*Dryocola clanedunensis* subsp. *sulfonylureivorans* (Li *et al.* 2024) Menasria *et al.* 2025	comb. nov. [basonym: *Cedecea sulfonylureivorans* Li *et al.* 2024]	006977	9
19	*Novosphingobium mangrovisedimenti* Heng 2025	nom. nov.†	006987	1
20	*Microbulbifer discodermiae* Moon and Park 2025	sp. nov.	006980	8
20	*Microbulbifer jejuensis* Moon and Park 2025	sp. nov.	006980	8
21	*Salinisphaera orenii* subsp. *orenii* (Park *et al.* 2012) Dif *et al.* 2025	comb. nov. [basonym: *Salinisphaera orenii* Park *et al.* 2012]	006975	12
21	*Salinisphaera orenii* subsp. *halophila* (Zhang *et al.* 2012) Dif *et al.* 2025	comb. nov. [basonym: *Salinisphaera halophila* Zhang *et al.* 2012]‡	006975	12
22	*Methylobacter arcticus* Rissanen *et al.* 2025	sp. nov.	006984	9

*The list editors have corrected the epithet to *latericius* (la.te.ri’ci.us. L. masc. adj. *latericius*, of brick, …). We thank H. Freese (Leibniz Institute DSMZ, Braunschweig) for alerting us to the error.

†Replacement name for the illegitimate species name *Novosphingobium mangrovi *Huang *et al.* 2023.

‡Erroneously given as subsp. nov. in the effective publication.

The following taxonomic opinions (i.e. the emendation of circumscriptions or the creation of synonyms) do not affect the status of a name under the International Code of Nomenclature of Prokaryotes. These taxonomic opinions can neither be considered as approved nor as disapproved by the International Committee on Systematics of Prokaryotes.

**Table:** 

Name/authors:	Article no.	Page no.
*Endobacterium* Menéndez *et al.* 2021 emend Menéndez *et al.* 2025*	006945	4

*The authors gave Menéndez *et al.* 2021 as the nomenclatural authority for the name.
